# Mechanochemically assisted hydrothermal synthesis of Sn-substituted MFI-type silicates

**DOI:** 10.1080/14686996.2018.1497404

**Published:** 2018-08-06

**Authors:** Kiyoshi Kanie, Moe Sakaguchi, Fumiya Muto, Mami Horie, Masafumi Nakaya, Toshiyuki Yokoi, Atsushi Muramatsu

**Affiliations:** a Institute of Multidisciplinary Research for Advanced Materials, Tohoku University, Sendai, Japan; b Institute of Innovative Research, Tokyo Institute of Technology, Yokohama, Japan; c JST-PREST, Tokyo, Japan; d JST-CREST, Tokyo, Japan

**Keywords:** Mechanochemical, zeolite, hydrothermal, silicate, MFI, 10 Engineering and Structural materials, 102 Porous / Nanoporous / Nanostructured materials, 301 Chemical syntheses / processing

## Abstract

Substitution of Al atoms in a zeolite framework by catalytic metal atoms has attracted considerable attention because the catalytic behavior can be tuned by the substituted atoms. In the present study, Sn-substituted MFI-type silicates were synthesized using a hydrothermal reaction of an amorphous Si-O-Sn precursor prepared by mechanochemical grinding of SiO_2_ and Sn(OH)_4_. The mechanochemical treatment was found to be a key technique for obtaining the amorphous Si-O-Sn precursor, where tetrahedral Sn^4+^ species were incorporated into the amorphous matrix. The Sn content in the framework of the MFI-type silicates was successfully controlled by the initial HCl/Si molar ratio of the hydrothermal procedures. Optical reflectance measurements revealed that the Sn^4+^ ions were dispersedly incorporated into the silicate framework while preserving the initial tetrahedrally coordinated species. Infrared results imply that the resulting Sn-substituted MFI-type silicate has Brønsted acid character. Precise control of the Brønsted and Lewis acid properties by Sn doping is a promising approach to the development of novel types of zeolite-based catalytic materials.

## GRAPHICAL ABSTRACT

**Figure:**
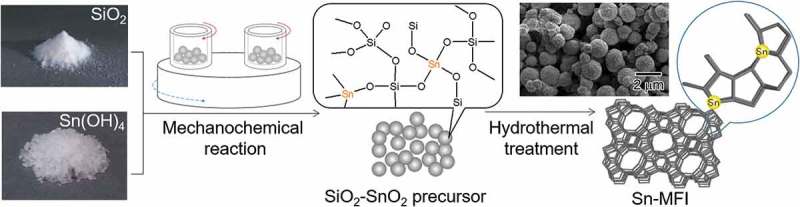

## 1. Introduction

Functional materials with hieratical structures have been attracted a great deal of attention in material science and technology because the unique structure-based functions widely applied as solar cells [1], biomedicines [2], two-dimensional nanoporous films [3], inverse opals [4], and membrane proteins [5]. Aluminosilicate zeolites, such as ZSM-5 and USY, widely used as adsorbents and catalysts, are representative materials with uniform hieratical micropore structures and strong acid sites. Recently, substitution of Al atoms in the zeolite framework by catalytic metal atoms has attracted considerable attention because the catalytic behavior can be tuned by the substituted atoms [6]. To date, diverse metal atoms (B, Ti, Fe, Ga, Ge, V, Sn, etc.) have been incorporated into the zeolite framework [7–11]. In particular, introduction of Sn^4+^ into the zeolite framework of MFI-type silicates leads to the introduction of Lewis acidic character, which can be used for catalysis in biomass conversion [12–15]. However, the introduction of a higher content of Sn^4+^ into the zeolite framework seems too difficult because of its large ionic radius and the low stability of the tetrahedral coordination. Furthermore, hydrothermal synthesis is a typical method for obtaining Sn-substituted silicate [16–18], however, formation of undesirable SnO_2_ on the extra-framework is a fundamental problem. Therefore, the development of a novel procedure for obtaining MFI-type silicates with a high Sn^4+^ content in the framework remains an important scientific goal [19–21]. As a new method, dealumination of silicates followed by the substitution of Sn^4+^ atoms is a unique and practical technique for obtaining tailor-made-type zeolites [22]. However, the method has a potential problem from the production of inactive Sn species, such as SnO_2_ on the extra-framework by the increase in the initial feeding amount of Sn^4+^ ions. To introduce the Sn atoms into the zeolite framework with higher Sn concentration, it is very important to create an Sn-O-Si bond in the initial step of Sn-silicate particle formation [19]. In our previous studies, we applied a mechanochemical treatment [23,24] to the preparation of titanosilicate zeolites. In this method, an amorphous Ti-O-Si composite is obtained by grinding TiO_2_ and SiO_2_ powders simultaneously with a planetary ball mill and converted into a titanosilicate zeolite through a subsequent hydrothermal treatment. The Ti atoms in the resulting titanosilicate are dispersedly incorporated into the silicate framework as a tetrahedrally coordinated species. The advantages of the mechanochemical reaction could be realized because the reaction promoted the formation of the amorphous Ti-O-Si precursor without formation of a crystalline TiO_2_ species by the mechanochemical energy provided by the collision and the friction of milling media. Notably, only TiO_2_ powders were not converted into an amorphous phase by the grinding in the absence of SiO_2_ powders. For the present research, we have focused our attention on the mechanochemical process for preparing Sn-silicate precursors with atomic-level mixing of Sn and Si so that the MFI-type Sn-substituted silicates with higher Sn content in the framework are synthesized using the subsequent hydrothermal procedure. As a result, 3.1 wt% of Sn atoms could be successfully introduced in the framework by optimizing the preparation conditions. We have also investigated the coordination structures and the solid acidic character of the Sn^4+^ atoms in the silicate frameworks using optical reflectance measurements.

## 2. Experimental details

### 2.1. Synthesis

Unless otherwise noted, all reagents were used as received. Water was doubly distilled, deionized, and filtered prior to use. As a Sn source, reagent-grade tin(IV) chloride pentahydrate (SnCl_4_ · 5H_2_O) was purchased from Wako Pure Chemicals Inc. (Osaka, Japan). Fumed silica Aerosil 200V (Nippon Aerosil, Tokyo, Japan) was chosen as the Si source of the mechanochemical step. The fumed silica was washed with water by filtration and dried at 120 °C prior to use. Carplex BS 304F (DSL Japan Co. Ltd., Tokyo, Japan) was used as the Si source of the following hydrothermal treatment. Aqueous 6.0 mol/L hydrochloric acid (HCl) and 2.5 mol/L NH_3_ solutions were obtained from Wako Pure Chemicals Inc. A 40 wt% tetrapropylammonium hydroxide (TPAOH) aqueous solution was purchased from Tokyo Chemical Industry Inc. Co. Ltd, Tokyo, Japan.

The representative procedure of the Sn-MFI zeolites via a mechanochemical route followed by a hydrothermal treatment is as follows: Initially, amorphous Sn(OH)_4_ for the mechanochemical reaction was prepared by dropwise addition of an aqueous NH_3_ solution (2.5 mol/L, 0.25 mL/min, total: 35 mL) into an aqueous SnCl_4_ · 5H_2_O solution (1.0 mol/L, 25 mL) with stirring at 0 °C. After the pH of the resulting mixture was adjusted to 2.0 by the addition of the NH_3_ solution, white-colored precipitates and the liquid phase were separated by centrifugation (15,000 rpm, 15 min), and the sediments were washed three times with ion-exchanged water by dispersal followed by centrifugation. The resulting solid precipitates were dried in an oven at 60 °C to obtain the amorphous Sn(OH)_4_ powder. A mixture of the amorphous Sn(OH)_4_ powder (0.30 g) and fumed silica (2.4 g) with a Si/Sn molar ratio of 20 was allowed to react mechanochemically through the milling using a planetary ball mill (Fritsch Inc. Co. Ltd., P-7, Idar-Oberstein, Germany) equipped with a silicon nitride pot and 7 balls (ϕ: 15 mm). The milling was conducted at autogenous temperature for 15 min followed by a pause for 15 min before another 15 min of milling to avoid the overheating of samples. The material was ground at 600 rpm for a total milling time of 0–24 h. To the resultant mixture with a milling time of 24 h, named g(SiO_2_–SnO_2_)_24_, a 40% of TPAOH aqueous solution was added as a structure direction agent (SDA) to crystallize the MFI-type silicate. The molar composition of the resulting mixture was adjusted to 1 SiO_2_: 0.2 SnO_2_: 0.5 TPAOH: 15 H_2_O: *x* HCl (*x* = 0.2–0.38) by the further addition of SiO_2_ (Carplex), ion-exchanged water, and an aqueous 6.0 mol/L HCl solution. The resulting white-colored gel-like mixture was well-mixed by stirring for 5 min at room temperature and hydrothermally treated in a Teflon-lined stainless autoclave at 160 °C for 120 h under stirring conditions (10 rpm). After the hydrothermal treatment, white-colored precipitates were collected by filtration. Then, the precipitates were washed five times with ion-exchanged water by dispersal followed by centrifugation. The product was dried at room temperature and calcined at 540 °C for 12 h under an air atmosphere to remove the SDA. The Sn-substituted MFI-type silicates Sn-S_MC_ thus obtained were characterized using the procedures shown in the following section.

For a control, a Sn-substituted MFI-type silicate Sn-S_HT_ was prepared via a conventional hydrothermal method [25] using SnCl_4_ · 5H_2_O and tetraethyl orthosilicate (TEOS, purchased from Tokyo Chemical Industry Inc. Co. Ltd.) as Sn and Si sources, respectively. The procedure was as follows: In a Teflon-made bottle, an aqueous 0.50 mol/L SnCl_4_ · 5H_2_O solution (0.68 mL) was diluted with ion-exchanged water (5.6 mL) at room temperature with stirring for 5 min. Then, a 40% of TPAOH aqueous solution (4.4 mL) was added to the solution and stirred for 10 min at room temperature. To the resulting mixture, an aqueous 6.0 mol/L HCl solution (0.60 mL) was added at the same temperature and stirred for 10 min. Then, the molar composition of the resulting mixture was adjusted to 1 SiO_2_: 0.2 SnO_2_: 0.5 TPAOH: 30 H_2_O: 0.19 HCl by the addition of TEOS (3.8 mL) at the same temperature. The precursor mixture thus obtained was stirred at 0 °C and room temperature for 6 and 42 h, respectively, and hydrothermally treated in a Teflon-lined stainless autoclave at 160 °C for 120 h under stirring conditions (10 rpm). Sn-S_HT_ was prepared as a white powder using the same procedure for the purification and then calcination of Sn-S_MC_.

### 2.2. Characterization

The powder X-ray diffraction (XRD) patterns were obtained on a Rigaku (Tokyo, Japan) Ultima-IV system equipped with a D/teX Ultra detector using CuKα radiation (40 kV, 40 mA). The UV–vis diffuse reflectance spectra were obtained using a Hitachi H-3900 spectrometer equipped with an integrating sphere attachment (Hitachi, Tokyo, Japan). The obtained reflectance spectra were converted into absorption using the Kubelka-Munk function [26]. The diffuse reflectance infrared Fourier transform (DRIFT) was measured with a Thermo Scientific NICOLET 6700 spectrometer equipped with an attenuated total reflectance system. Fourier transform infrared (FTIR) spectra were measured on a Varian FTS7000 spectrometer using KBr pellets (Varian, CA, USA). The specific surface area and the micropore volume were estimated based on the nitrogen adsorption isotherm collected using a Bel Japan Belsorp miniII apparatus (Bel Japan, Osaka, Japan). The particle size and shape of the materials were evaluated using a JEOL (Tokyo, Japan) JSM7800-F field emission scanning electron microscope (FE-SEM). Inductively coupled plasma atomic emission spectroscopy (ICP-AES) measurements were performed on a SPECTRO ARCOS system (SPECTRO Analytical Ins., Kleve, Germany). X-ray photoelectron spectroscopy (XPS) data were collected using a PHI 5600 ESCA system and MgKα radiation (Physical Electronics Inc., MN, USA). X-ray fluorescence (XRF) analysis was carried out using a Rigaku ZSX Primus II system. The amounts of Al and Y contaminant ions in g(SiO_2_–SnO_2_)_24_ introduced by mechanochemical grinding using Si_3_N_4_ pot and balls were determined by semi-quantitative (SQX) analysis of the XRF measurements.

## 3. Results and discussion

**Figure 1.**
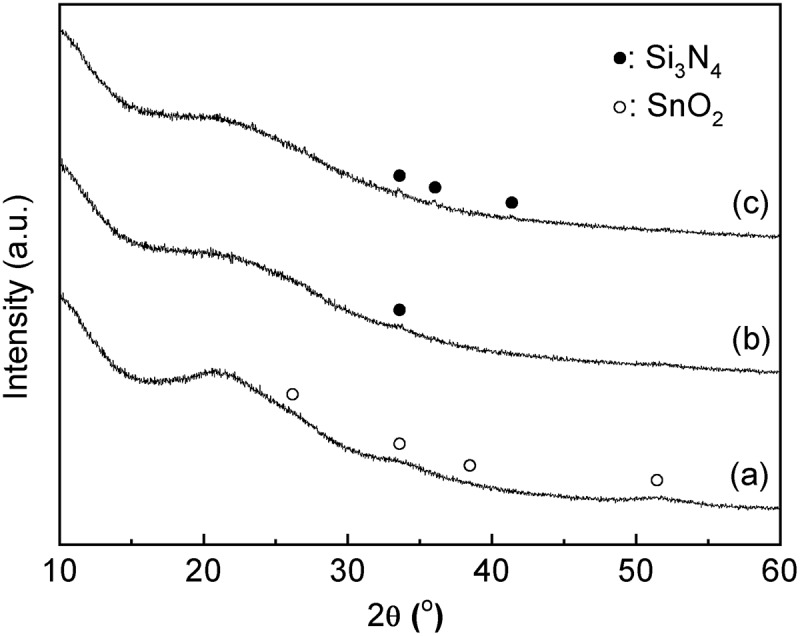
XRD patterns of a mixture of the amorphous Sn(OH)_4_ powder and fumed silica for the Si/Sn molar ratio of 20; (a) before the mechanochemical treatment; (b) after grinding for 12 h, g(SiO_2_–SnO_2_)_12_; (c) after grinding for 24 h, g(SiO_2_–SnO_2_)_24_ (all patterns are vertically offset for clarity).

**Figure 2.**
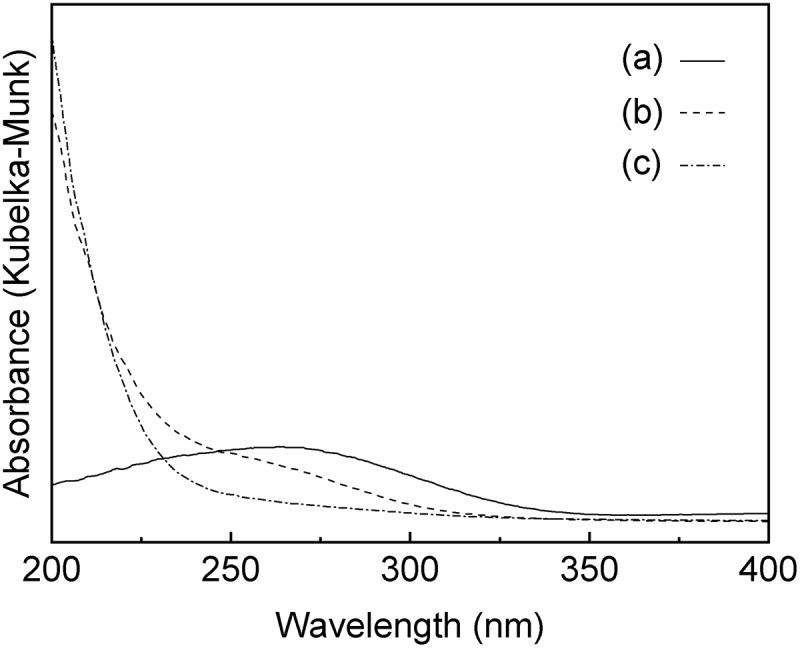
UV-vis spectra of a mixture of the amorphous Sn(OH)_4_ powder and fumed silica for the Si/Sn molar ratio of 20; (a) before the mechanochemical treatment; (b) after grinding for 12 h, g(SiO_2_–SnO_2_)_12_; (c) after grinding for 24 h, g(SiO_2_–SnO_2_)_24._

**Figure 3.**
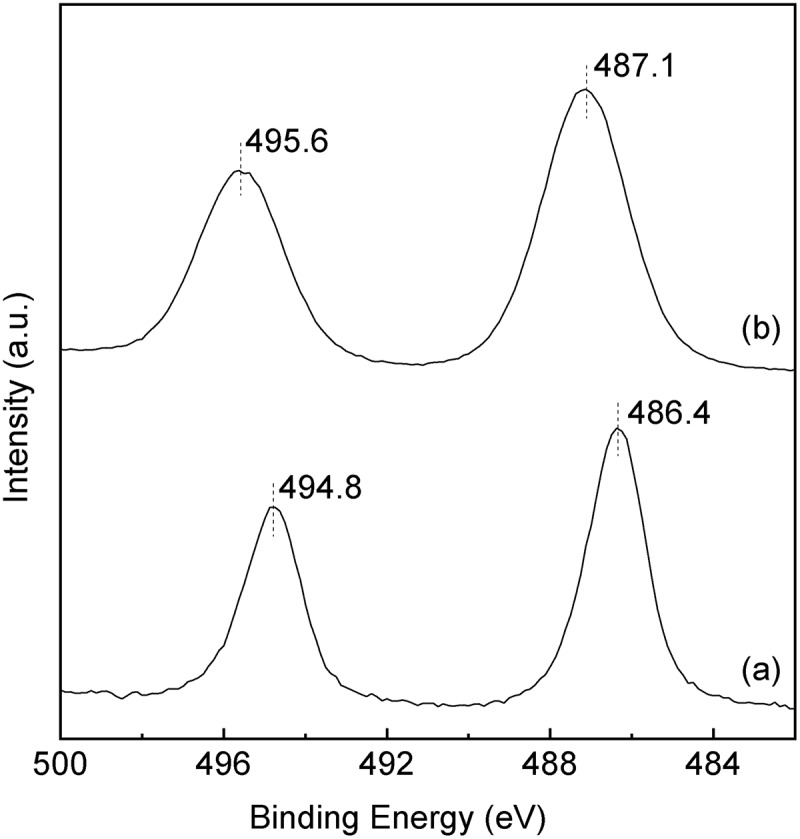
Sn 3d_3/2_ and 3d_5/2_ XPS spectra of (a) before the mechanochemical treatment; (b) after grinding for 24 h, g(SiO_2_–SnO_2_)_24._

Figures 1 and 2 show the XRD patterns and the UV-vis spectra of the SnO_2_-SiO_2_ precursor before and after the mechanochemical treatment, respectively. Before the treatment, a broad halo derived from an amorphous mixture of Sn(OH)_4_ SiO_2_ is observed at approximately 21° (Figure 1(a)). Broad peaks due to crystalline SnO_2_ formation are also slightly apparent in the diffraction pattern. After the grinding at 600 rpm for 12 h, these peaks assigned as SnO_2_ crystals disappear (Figure 1(b)), suggesting that the crystalline SnO_2_ degraded and formed an amorphous Sn-O-Si mixture due to the mechanochemical grinding. As shown in Figure 1(c), weak peaks at 33.6°, 36.0°, and 41.4°, assignable to β-Si_3_N_4_ derived from the milling pot and balls, are slightly apparent after the grinding for 24 h. The UV-vis spectrum of the SnO_2_-SiO_2_ precursor before grinding exhibited a broad absorption band at approximately 260 nm (Figure 2(a)). The absorption behavior is consistent with a crystalline SnO_2_ powder with octahedral Sn^4+^ spaces [22,27]. On the other hand, a broad and strong absorption at approximately 200 nm is observed by the grinding for 12 h, and the absorption band at approximately 260 nm before grinding is shifted to a lower wavelength (Figure 2(b)). After the grinding for 24 h, the absorption band at approximately 260 nm due to the crystalline SnO_2_ completely disappeared (Figure 2(c)), and only a strong absorption at approximately 200 nm could be ascribed to tetrahedral Sn^4+^ spaces, as seen. Figure 3 shows the Sn 3d_3/2_ and Sn 3d_5/2_ XPS spectra before and after grinding for 24 h. The mixture of SiO_2_ and Sn(OH)_4_ before the mechanochemical reaction showed signals at 494.8 and 486.4 eV, which match octahedral Sn^4+^ species [28,29]. On the other hand, Sn species in the SiO_2_-SnO_2_ precursor after the mechanochemical reaction for 24 h have the higher binding energies of 495.6 and 487.1 eV, which indicates that tetrahedral Sn^4+^ species are present in the mixture [28,29]. These characterization results reveal that the Sn(OH)_4_ powder is mechanochemically reacted with fumed silica in the solid phase during the grinding for 24 h, forming Sn-O-Si bonding with tetrahedral Sn^4+^ species incorporated into the amorphous matrix. Here, utilization of commercially available SnO_2_ powder as the Sn^4+^ source was not effective to prepare the SiO_2_-SnO_2_ amorphous precursor because XRD diffraction peaks due to crystalline SnO_2_ were still remained after the 24 h grinding. XRF measurements of the g(SiO_2_–SnO_2_)_24_ precursor revealed that the amounts of Al and Y contaminant ions by the mechanochemical grinding using the Si_3_N_4_ pot and balls were 0.06 wt% and 0.04 wt%, respectively. The molar ratio of Sn/Al and Sn/Y were calculated as 17 and 75, respectively. The trace amount of the Al and Y ions is not fundamentally influenced to the following MFI-zeolite synthesis and the resulting acid characters.

**Figure 4.**
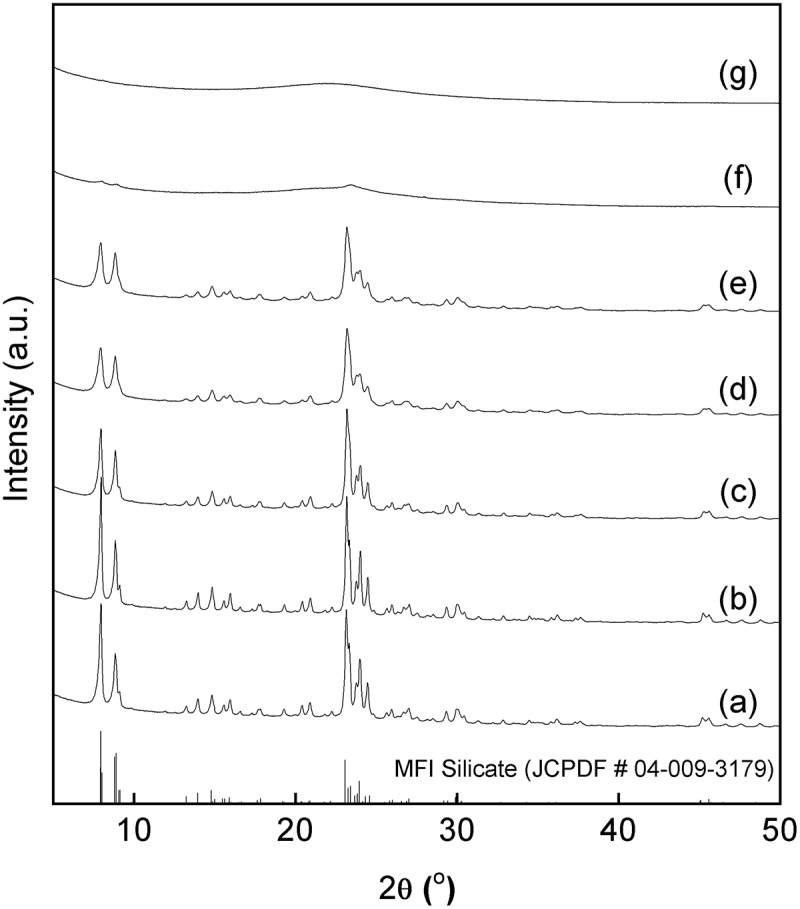
XRD patterns of Sn-MFI silicates Sn-S_MC_ obtained by changing the HCl/Si molar ratio: (a) 0.20; (b) 0.25; (c) 0.30; (d) 0.35; (e) 0.36; (f) 0.37; (g) 0.38 (all patterns are vertically offset for clarity).

Figure 4 shows XRD patterns of solid particles obtained by the hydrothermal treatment of the mechanochemically prepared g(SiO_2_–SnO_2_)_24_ precursor in the presence of TPAOH. The hydrothermal conditions were fixed at 160 °C for 120 h, and the HCl/Si molar ratio was varied from 0.20 to 0.38 to evaluate the effect of chloride ions on the Sn-MFI synthesis. In this range of the HCl/Si molar ratio, pH value was predominantly controlled by the addition amount of TPAOH. All the samples were calcined at 540 °C for 24 h in air before the XRD measurement. From the XRD patterns of samples prepared with HCl/Si ≤ 0.36, all diffraction peaks can be assigned to the MFI-type crystal structure (JCPDF # 04–009-3179, Figure 4(a–e)). The half-widths of the diffraction peaks increased with increasing HCl/Si ratio, which means a decrease in the primary particle size of Sn-S_MC_. On the other hand, only amorphous solids were formed for HCl/Si = 0.37 and 0.38. The positive effect of the chloride concentration on the control of the doping amount of the Sn in the framework of the MFI-type silicates is mentioned below. However, the excessive increase in the HCl/Si ratio suppressed the formation of the desired Sn-S_MC_.

**Figure 5.**
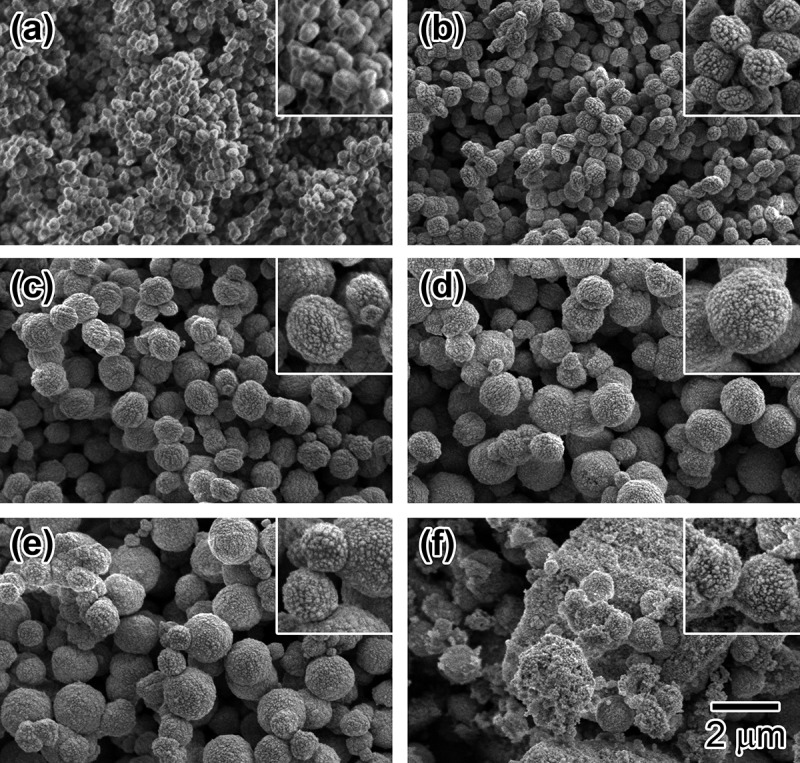
SEM images of Sn-MFI prepared with various HCl/Si molar ratios: (a) 0.20; (b) 0.25; (c) 0.30; (d) 0.35; (e) 0.36; (f) 0.37. The scale bar shown in (f) is common for all images. The insets are 2-fold magnified images for observing the surface roughness of the solid particles.

Figure 5 shows SEM images of the solid particles prepared with HCl/Si = 0.2–0.37. All particles except for those of HCl/Si = 0.36 (Figure 5(e)) have basically a sphere-shaped morphology with rough surfaces. The particle mean diameter was increased by the increase in the HCl/Si ratio from 0.20 to 0.36. As shown in Figure 4, the primary particle size decreased with an increasing HCl/Si ratio. This result suggests that the sphere-shaped particles are the aggregated secondary polycrystalline particles. From the XRD pattern shown in Figure 4(f), the solid particles obtained with HCl/Si = 0.37 have an amorphous structure. However, from the SEM image (Figure 5 (f)), some sphere-shaped particles with rough surfaces are seen in the irregular shaped solid matrix, probably due to partial crystallization.

**Figure 6.**
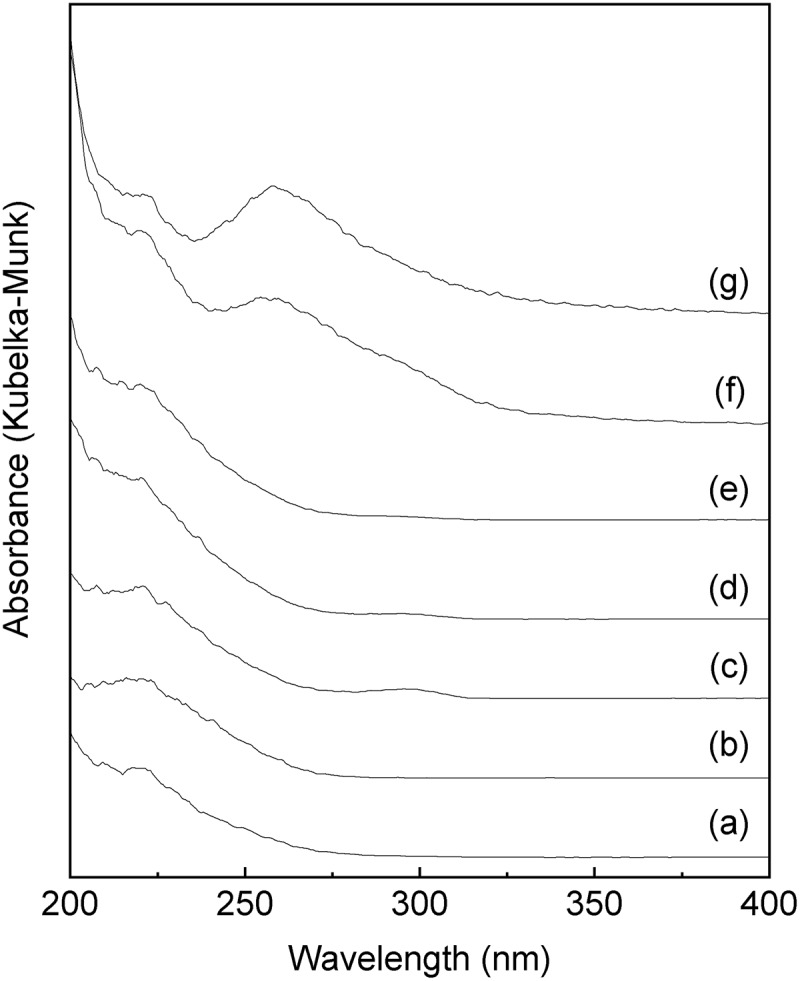
UV-vis spectra of Sn-MFI silicates Sn-S_MC_ prepared at various HCl/Si molar ratios: (a) 0.20; (b) 0.25; (c) 0.30; (d) 0.35; (e) 0.36; (f) 0.37; (g) 0.38 (all spectra are vertically offset for clarity).

**Figure 7.**
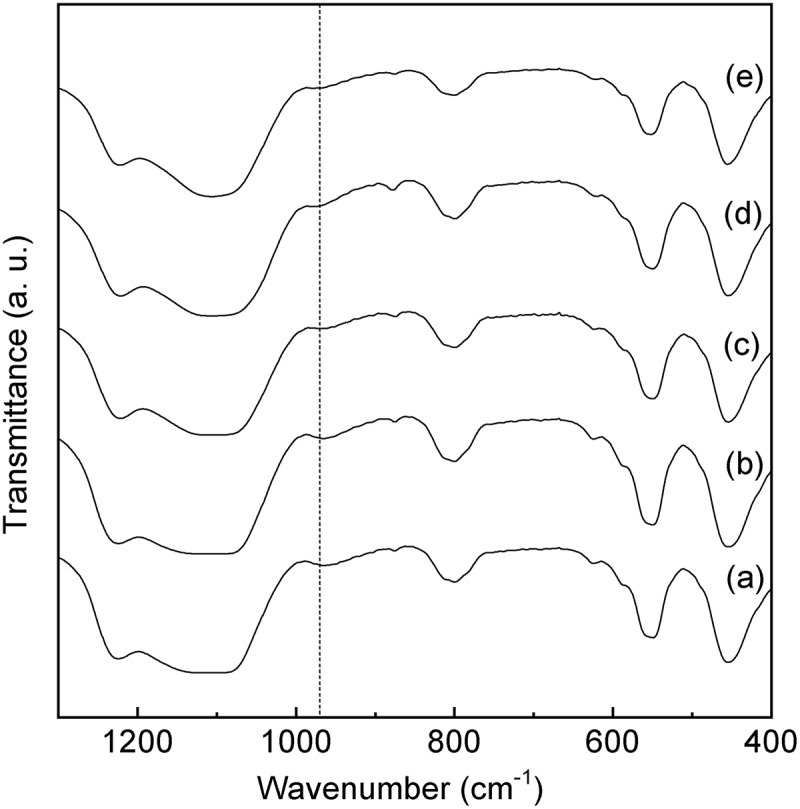
FTIR spectra of Sn-MFI silicates Sn-S_MC_ obtained at various HCl/Si molar ratios: (a) 0.20; (b) 0.25; (c) 0.30; (d) 0.35; (e) 0.36 (all spectra are vertically offset for clarity).

Figure 6 shows the UV-vis spectra of the Sn-MFI silicates with various HCl/Si molar ratios. The samples with an Sn-MFI silicate crystal structure (Figure 6(a–e)) exhibit a broad absorption band at approximately 220 nm. Notably, the absorption band at approximately 260 nm that is attributed to octahedral Sn^4+^ spaces is not observed for the calcined Sn-S_MC_. On the other hand, the band at 240–250 nm is still in debate, however, penta-coordinated Sn species [30,31], extra-framework Sn species [17], and small tin oxide particles [30,32] have been assigned to the peaks at around 240–250 nm. These results strongly support the idea that Sn^4+^ ions were introduced into the Sn-MFI framework as tetrahedrally coordinated states. In contrast, a broad and strong absorption at approximately 260 nm, due to the formation of the octahedral Sn^4+^ spaces, is clearly observed for the calcined amorphous powders (Figure 6(f) and (g)). Furthermore, the Sn-MFI silicates Sn-S_MC_ were characterized using FTIR measurements (Figure 7). An absorption at 980 cm^−1^ due to Sn-O-Si bonding is clearly observed for all samples [27,33]. These findings indicate that Sn^4+^ ions are incorporated in the Sn-MFI silicate framework as tetrahedrally coordinated states.

The Sn content, Brunauer–Emmett–Teller (BET) surface area (*S*_BET_), external surface area (*S*_ext_), total volume (*V*_total_), micropore volume (*V*_micro_), average particle diameter (*d*_ave_), calculated particle diameter (*d*_cal_), and crystallite size of Sn-S_MC_ are listed in Table 1. The Sn content was determined using ICP-AES measurement, and surface areas and volumes were measured using the nitrogen adsorption method. Note that the Sn content increased from 1.9 to 3.1 wt% as the HCl/Si ratio was increased from 0.20 to 0.36. As mentioned above, further increase in the HCl/Si ratio led to the formation of the amorphous phase. This result suggests that the maximum doping amount of Sn in the Sn-S_MC_ framework is 3.1 wt% by way of the mechanochemical route. The *S*_BET_ tends to slightly expand with increasing HCl/Si ratio. On the other hand, *S*_ext_ was strongly affected by the HCl/Si ratio and drastically increased to 86 m^2^/g at HCl/Si = 0.36. Such behavior might originate from surface roughening in Sn-S_MC_ by chloride ions. It was reported that the addition of chloride ions in the hydrothermal synthesis of inorganic particles induced heterogeneous nucleation on the growing surfaces, yielding polycrystalline particles with rough surfaces [34]. Here, the crystallite size of Sn-S_MC_, calculated from the half-width of the (110) diffraction peak using the Scherrer equation, decreased with an increase in the HCl/Si ratio. In the present system, the increase in the doping amount of Sn in the Sn-S_MC_ framework led to the formation of cracks and boundaries in the Sn-S_MC_ crystal structure that reduced the crystallite size. An increase in the roughness of the particle surfaces and in the Sn concentration in the Sn-S_MC_ framework are the most plausible explanations for the decrease in the crystallite size of Sn-S_MC_. The large difference between *D*_ave_ and the crystallite size of Sn-S_MC_ also suggests that the present Sn-S_MC_ particles have polycrystalline structures. *V*_micro_ is unaffected by the HCl/Si ratio, whereas *V*_total_ increased as the HCl/Si ratio was increased from 0.25 to 0.36. This behavior implies that the volume of the mesopores in the Sn-S_MC_ particles increased with increasing HCl/Si concentration, which is a positive effect for the application as adsorbents. The *V*_total_ values are sufficiently large to demonstrate the microporous MFI-type structure of Sn-S_MC_.

**Table 1.** The Sn content, BET surface area, and the external surface area of Sn-S_MC._

HCl/Si	Sn content (wt%)	*S*_BET_ (m^2^/g)	*S*_ext_ (m^2^/g)	*V*_total_ (mL/g)	*V*_micro_ (mL/g)	*d*_ave_^a^ (nm)	Crystallite size (nm)^b^
0.20	1.9	462	26	0.31	0.17	380	61
0.25	2.0	454	4	0.24	0.19	610	60
0.30	2.5	459	11	0.23	0.18	940	49
0.35	3.1	495	79	0.28	0.18	1180	32
0.36	3.1	527	86	0.31	0.19	1080	32

^a^Particle diameters determined by averaging over more than 130 particles in SEM images (Figure 5). ^b^Calculated with the Scherrer equation from the half-width of the XRD patterns shown in Figure 4.

**Figure 8.**
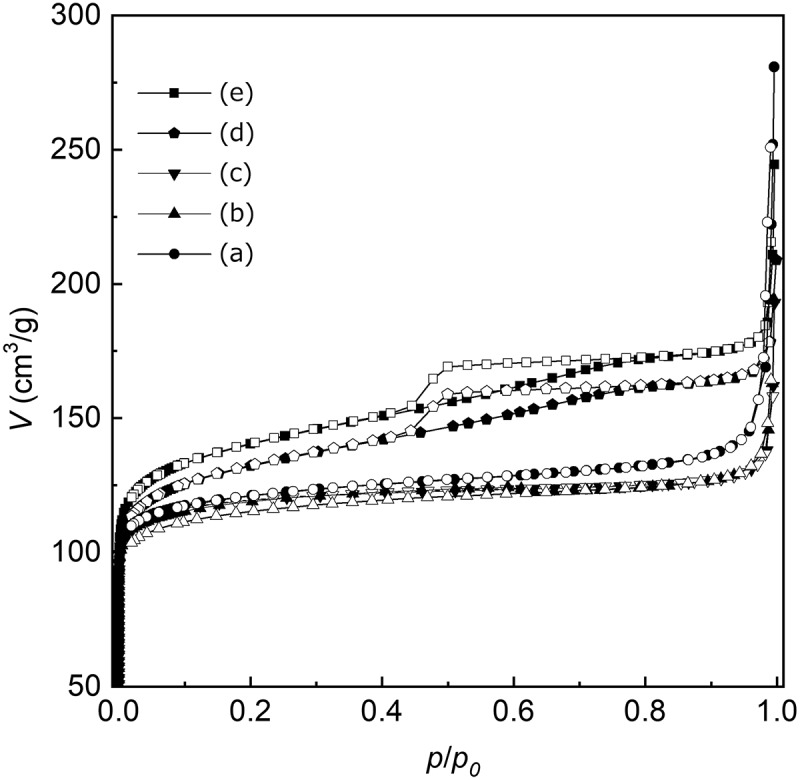
Nitrogen sorption isotherms of Sn-MFI silicates Sn-S_MC_ obtained at various HCl/Si molar ratios: (a) 0.20; (b) 0.25; (c) 0.30; (d) 0.35; (e) 0.36.

*V*_total_ increased as the external surface area expanded. In nitrogen sorption isotherms of Sn-MFI (HCl/Si ≥ 0.35), an N_2_ hysteresis loop was observed, which suggests that an increase in the external surface area is caused by the introduction of mesoporosity into Sn-MFI particles (Figure 8).

**Figure 9.**
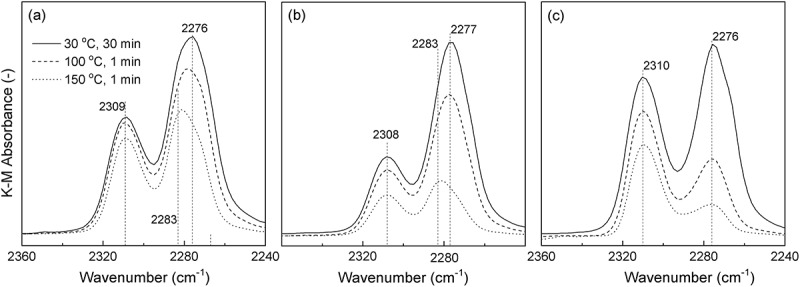
FTIR spectra using CD_3_CN as a probe molecule for (a) Sn-S_MC_ (HCl/Si = 0.20); (b) Sn-S_MC_ (HCl/Si = 0.36); (c) Sn-S_HT_ (HCl/Si = 0.19).

Figure 9 shows the FTIR spectra of CD_3_CN dosed onto Sn-S_MC_ and Sn-S_HT._ The profiles were taken using the DRIFTS method. The background profiles of the spectra were corrected at room temperature by the samples after heating at 550 °C for 1 h under a He atmosphere. Adsorption of CD_3_CN molecules on the Sn-S_MC_ and Sn-S_HT_ surfaces were performed as follows: Initially, the CD_3_CN probe molecules were heated at 120 °C, and then a large excess of gas molecules was introduced into an Sn-S_MC_ and Sn-S_HT_ mounted measurement chamber at 30 °C. The solid lines in Figure 9 exhibit the profiles taken after 30 min for the adsorption at 30 °C. Dashed and dotted lines demonstrate the spectra after heating at 100 and 150 °C, respectively, for 1 min to eliminate the CD_3_CN probe molecules. All the profiles were collected after cooling to 30 °C. As exhibited in Figure 9, all the samples show an absorption at approximately 2309 cm^−1^, which is attributable to C ≡ N stretching of CD_3_CN molecules adsorbed on the Sn-site of Sn-S_MC_ and Sn-S_HT_ in the framework. Montejo-Valencia et al. reported that an Sn-doped BEA zeolite has ‘closed’ and ‘open’ Sn sites with Sn(OSi)_4_ and Sn(OSi)_3_(OH) structures, respectively [30,35]. The absorbance peaks of CD_3_CN on the ‘closed Sn’ and the ‘open Sn’ sites are at 2309 cm^−1^ and 2317 cm^−1^, respectively. This result suggests that Sn-S_MC_ and Sn-S_HT_ have the ‘closed’ Sn(OSi)_4_ structure [31,32]. Furthermore, all samples show no absorbance at 2317 cm^−1^ due to the ‘open’ Sn site. The absorbance at approximately 2276 cm^−1^ can be assigned to the CD_3_CN molecules adsorbed on the silanol moieties of Sn-S_MC_ and Sn-S_HT_. For Sn-S_HT_, the absorbance peak simply decreased with the increase in the heat treatment temperature. However, for Sn-S_MC_, not only a decrease in absorbance but also the appearance of a new absorbance at approximately 2283 cm^−1^ are observed. This difference in spectra is possibly due to the difference in the packing structure of the CD_3_CN molecules between on the surfaces of Sn-S_MC_ and Sn-S_HT_ [36,37]. As listed in Table 1, the Sn content of Sn-S_MC_ (HCl/Si = 0.20) is lower than that of Sn-S_MC_ (HCl/Si = 0.36), however, the ratio of absorbance: 2309 cm^−1^/2276 cm^−1^ of Sn-S_MC_ (HCl/Si = 0.20) is higher than that of Sn-S_MC_ (HCl/Si = 0.36). This behavior is probably due to a larger *S*_ext_ for Sn-S_MC_ (HCl/Si = 0.36) than for Sn-S_MC_ (HCl/Si = 0.20) because the amount of silanol moieties, absorbable to CD_3_CN, depends on the *S*_ext_ of the silicates. To date, there is no report on the new absorbance at approximately 2283 cm^−1^, only observed for the samples obtained by way of the present mechanochemical method. However, there is one possibility due to the absorbance of the CD_3_CN molecules adsorbed on the Brønsted acid sites through hydrogen bonding formed by the Sn ion doping. Thus, observed Brønsted acid sites might be due to the proton of the silanol group near the Sn atoms [38,39]. For example, Yang et al. calculated the acid strength of the Brønsted acid sites in metal-doped MFI-silicates using density functional theory [40]. Their result suggested that the resulting silicates have a Brønsted acid nature, and the acidity increased as follows: MFI << Ge < Zr = B < Pb < Sn < Al. Furthermore, although the Brønsted acidity of Zr^4+^ is lower than that of Sn^4+^, a Zr-doped BEA silicate exhibits a Brønsted acid property [41]. From these results, there is a large possibility that the present Sn-S_MC_ has Brønsted acid character, and the new absorbance at approximately 2283 cm^−1^ originates from the CD_3_CN molecules located at the Brønsted acid sites in Sn-S_MC_.

## 4. Conclusions

Sn-substituted MFI-type silicates were successfully prepared using a hydrothermal reaction of an amorphous Si-O-Sn precursor prepared using a mechanochemical grinding of fumed SiO_2_ and Sn(OH)_4_. The mechanochemical treatment at 600 rpm for 24 h was found to be a key and powerful technique for obtaining the amorphous Si-O-Sn precursor, where tetrahedral Sn^4+^ species were incorporated into the amorphous matrix. The Sn content in the silicates increased from 1.9 to 3.1 wt% as the HCl/Si molar ratio was increased from 0.20 to 0.36. Further increase in the HCl/Si molar ratio resulted in the formation of an amorphous solid. UV-vis and FTIR measurements revealed that the Sn^4+^ ions were dispersedly incorporated into the silicate framework as a tetrahedrally coordinated species. From the FTIR investigation using the DRIFTS method, there is a large possibility that the present Sn-S_MC_ has Brønsted acid character. Precise control of the Brønsted and Lewis acid properties by Sn doping is a promising approach for the development of novel types of zeolite-based catalytic materials.
